# Long Non-coding RNAMALAT1 Knockdown Alleviates Cerebral Ischemia/Reperfusion Injury of Rats Through Regulating the miR-375/PDE4D Axis

**DOI:** 10.3389/fneur.2020.578765

**Published:** 2021-01-14

**Authors:** Guangjian Zhang, Qingdong Wang, Daoqing Su, Yingliang Xie

**Affiliations:** ^1^Department of Neurosurgery, Weifang People's Hospital, Weifang, China; ^2^Department of Neurology, Liaocheng People's Hospital, Liaocheng Hospital Affiliated to Shandong First Medical University, Liaocheng, China; ^3^Department of Neurosurgery, Liaocheng People's Hospital and Liaocheng Brain Hospital, Liaocheng Hospital Affiliated to Shandong First Medical University, Liaocheng, China

**Keywords:** cerebral ischemia/reperfusion injury, lncRNA MALAT1, miR-375, PDE4D, neurosurgery

## Abstract

**Objectives:** Cerebral ischemic/reperfusion injury (CI/RI) is the clinical manifestation of cerebral ischemic stroke, which severely affects the health and life of the patients. We aimed to investigate the regulatory mechanism of long non-coding RNA (lncRNA) metastasis-associated lung adenocarcinoma transcript 1 (MALAT1) on CI/RI in this study.

**Methods:** The expression of lncRNA MALAT1 and miR-375 was detected by qRT-PCR. MTT was utilized to measure the viability of PC-12 cells. The levels of lactate dehydrogenase (LDH), superoxide dismutase (SOD), and reactive oxygen species (ROS) were detected by LDH assay, SOD assay, and ROS assay, respectively. The apoptosis rate of PC-12 cells was measured by flow cytometry analysis. Through enzyme-linked immunosorbent assay, the levels of NF-α, IL-1β, and IL-6 were determined. The interactions between miR-375 and MALAT1/PDE4D were predicted by Starbase/Targetscan software and verified by the dual-luciferase reporter assay. Western blot assay was performed to determine the protein expression of Bcl-2, Caspase-3, and PDE4D.

**Results:** LncRNA MALAT1 expression was highly upregulated in the middle cerebral artery occlusion (MCAO)/reperfusion (R) model of rats. Both MALAT1 downregulation and miR-375 upregulation reversed the inhibitory effect of oxygen and glucose deprivation (OGD)/R on cell viability and the promoting effects on LDH level, cell apoptosis, and inflammatory factors levels. MALAT1 targeted miR-375, whereas miR-375 targeted PDE4D. Overexpression of miR-375 attenuated OGD/R-induced injury in PC-12 cells by targeting PDE4D. Both the low expression of miR-375 and high expression of PDE4D reversed the promoting effect of MALAT1 knockdown on SOD level and the inhibitory effects on ROS level, inflammatory factor levels, and cell apoptosis.

**Conclusion:** Suppression of MALAT1 alleviates CI/RI of rats through regulating the miR-375/PDE4D axis. This study provides a possible therapeutic strategy for human CI/RI in clinic.

## Introduction

Cerebral ischemic/reperfusion injury (CI/RI) caused by brain ischemia and the following blood supply is a common cerebrovascular disease (1, 2). Acute cerebral ischemic/reperfusion may even induce rupture of blood vessels or cause extensive edema around the lesion (3). Nowadays, although the tissue plasminogen activator is considered to be the most effective therapy for treating CI/RI (4), some side effects remain to be faced, such as the secondary hemorrhage risk (5). In consequence, further molecular research on the potential mechanism of CI/RI and developing potential therapeutic targets in CI/RI are the urgent problems to be solved.

Recently, researchers have found that long non-coding RNAs (lncRNAs) are important regulators in CI/RI (6–8). For instance, upregulation of AK038897 aggravates CI/RI in a mouse model (6). RASD1 downregulation attenuates CI/RI of rats (7). SNHG14 knockdown restrains inflammatory factor release of adrenal pheochromocytoma cells induced by cerebral ischemia/reperfusion (CI/R), thus alleviating CI/RI *in vitro* (8). Notably, emerging evidences have displayed that MALAT1 is involved in modulating renal ischemia/reperfusion injury (9) or myocardial ischemia/reperfusion injury (10). More importantly, MALAT1 has been reported to protect brain microvascular endothelial cells against oxygen and glucose deprivation (OGD) (11). However, the detailed mechanism of MALAT1 regulating CI/RI *in vivo* and *in vitro* has not been fully elucidated.

MicroRNAs (miRNAs) exhibit their suppressive roles on CI/RI (12–14). Wang et al. have uncovered that overexpression of miR-22 promotes formations of cerebrovascular and cranial nerves, thus attenuating CI/RI in a rat middle cerebral artery occlusion (MCAO)/reperfusion (R) model (12). Chen et al. have demonstrated that in models of rats, miR-193b serves as a protector against CI/RI and can diminish infarct size and pathomorphological changes of neuron (13). Lu et al. have revealed that miR-219a-5p upregulation elevates cell viability and restrains lactate dehydrogenase (LDH) levels and the apoptosis rate of cells, eventually allaying CI/RI *in vitro* (14). In addition, recent studies have reported that miR-375 overexpression can also mitigate CI/RI *in vitro* or *in vivo* (7, 15). Wang et al. have confirmed that upregulation of miR-375 is helpful in improving CI/RI of rats (7). Ou et al. have presented that both *in vitro* and *in vivo*, miR-375 plays a protective role in CI/RI (15). However, we are still unclear whether the protective effect of miR-375 on CI/RI is modulated by MALAT1.

Phosphodiesterase 4D (PDE4D), a kind of enzyme from the cAMP-specific PDE family, is mainly expressed in many cell types, such as central nervous cells and immune cells (16, 17). PDE4D is known as a major risk factor gene for ischemic stroke (IS). For instance, the polymorphisms of the PDE4D gene (SNP83) have been reported to be associated with the risk of IS in the young Chinese population (18). More importantly, PDE4D inhibition is believed to restrict neuroinflammation during IS (19). In addition, PDE4D has been found to serve as an oncogene regulated by miRNAs to promote tumor progression in various human cancers, such as miR-494-PDE4D in gastric cancer (20) and miR-203a-3p-PDE4D and miR-139-5p-PDE4D in colorectal cancer (21, 22). Notably, a recent study has been demonstrated that miR-219-5p can target PDE4D to attenuate CI/RI (14). However, whether PDE4D is regulated by miR-375 to be involved in CI/RI and its underlying mechanism are still unknown.

In this study, the effects of MALAT1 on cell viability, LDH levels, cell apoptosis and inflammatory factor release, and the regulatory mechanism between MALAT1 and miR-375/PDE4D on OGD/R injury were investigated. Our results are aimed to provide a potential strategy for attenuating injury against CI/R in clinical trials.

## Materials and Methods

### Construction of CI/RI Rats Models

The healthy male Sprague–Dawley (SD) rats (weighing 220 ± 20 g) were procured from GDMLAC (Medical Laboratory Animal Center of Guang Dong, Guangzhou, China) and fed freely under a 12-h cycle (12 h for light and 12 h for dark) at 20°C. Rats were divided into two groups: the MCAO group and the sham group (*n* = 5). The CI/RI rat model was established by MCAO based on the previous study (23). In brief, rats in the MCAO group were anesthetized by pentobarbital sodium (50 mg/kg). Under the operating microscope, a midline incision was made to expose the right common carotid artery, external carotid artery, and internal carotid artery. MCAO was induced through advancing a surgical nylon monofilament by a rounded tip into the lumen of the internal carotid artery gently from the right external carotid artery until the rounded tip blocked the origin of the middle cerebral artery. After MCAO for 1 h, blood reperfusion at different time points (12, 24, and 48 h) was performed to induce construction of the CI/RI model. The rats in the sham group underwent the same surgical procedure except MCAO. This study was conducted after obtaining the approval of Laboratory Animal Ethics Committee of Weifang People's Hospital (No. 20200601), and we confirmed that all experiments were performed following experimental animal management and animal welfare at the Branch of Weifang People's Hospital.

### Cell Grouping, Transfection, and Establishment of the OGD/R Model

The rat adrenal pheochromocytoma cell line (PC-12) was procured from Xinyu Biotech, Ltd (Shanghai, China), and cultured in DMEM containing 10% fetal bovine serum (FBS) and 0.1% penicillin/streptomycin at 37°C with 5% CO_2_. The cells were divided into two groups: the OGD/R group and the control group. For the OGD/R group, the cells were cultured in glucose-free DMEM at 37°C with 5% CO_2_ and 95% N_2_ (simulation of cerebral ischemia) for 3 h. After that, the cells were exposed in a normoxic environment (simulation of reperfusion) with 95% air and 5% CO_2_ at different time points (12, 24, and 48 h) to induce the OGD/R model. The cells in the control group were treated without OGD.

The shRNA-negative control (sh-NC) and shRNA-MALAT1-1/-2 (sh-MALAT1-1/-2) were procured from Sangon Biotech, Inc (Shanghai, China). Overexpression-MALAT1 (pcDNA-MALAT1), overexpression-PDE4D (pcDNA-PDE4D) and their negative control (pcDNA-NC), miR-375 mimics, miR-375 inhibitor, and their negative control (miR-NC) were all obtained from Ribo Biotech, Ltd (Guangzhou, China). Using the Lipofectamine RNAiMAX kit (Invitrogen, Carlsbad, CA, USA), the aforementioned agents were transfected into the OGD/R-induced cells for 48 h. After then, the cells were harvested to perform the following experiments.

### Quantitative Reverse-Transcription PCR (qRT-PCR)

Total RNA was extracted from cells and brain tissues of rats using TRIzol kit (Invitrogen, Inc.) and then reversely transcribed into cDNA by the GoScript reverse transcription system (Promega, Madison, WI, USA). The reaction conditions were 94°C for 10 min, followed by 40 cycles at 94°C for 10 sec, 60°C for 20 sec, and 72°C for 1 min. GADPH or U6 was used as the internal reference. The primer sequences were MALAT1 forward primer, 5′-AGGCAGGTGGGAGATGAT-3′, reverse primer, 5′-GGTCTGTGCTAGATCAAAAGGC-3′; miR-375 forward primer, 5′-ACACTCCAGCTGGGTTTGTTCGTTCGGCTCGC-3′, reverse primer, 5′-CTCAACTGGTGTCGTGGAGTCGGCAATTCAGTTGAGTCACGCGA-3′;

PDE4D forward primer, 5′-TTTTGCCAGTGCAATACATGATG-3′, reverse primer, 5′-CAGAGCGAGTTCCGAGTTTGT-3′; GAPDH forward primer, 5′-GGTGGTCTCCTCTGACTTCAACA-3′; reverse primer, 5′-GTTGCTGTAGCCAAATTCGTTGT-3′; U6 forward primer, 5′-CTCGCTTC-3′; reverse primer, 5′-AACGCTTCACGAATTTGCGT-3′. Gene expression was quantified using the 2^−ΔΔ*Ct*^ method.

### Superoxide Dismutase (SOD) and Reactive Oxygen Species (ROS) Detection Assays

According to manufacturer protocol, the levels of SOD/ROS in PC-12 cells or brain tissues of rats were measured using Superoxide Dismutase Activity Assay Kit or Reactive Oxygen Species Assay Kit (Abcam, Cambridge, MA, USA), respectively. SOS activity (OD450) and ROS fluorescence (OD520) were measured using a Synergy HT microplate reader (Biotek, Winooski, VT, USA).

### MTT Assay

The viability of PC-12 cells was detected by MTT assay. The OGD/R-induced PC-12 cells were seeded into a 96-well plate with 2 × 10^5^ cells per well incubating for 24 h at 37°C, followed by adding 20 μl MTT (GENECHEM, Inc, Shanghai, China) to each well. After 2 h of incubation at 37°C, the viability (OD450) was analyzed by a Multiskan Spectrum microplate reader (Thermo Fisher Scientific, Waltham, MA, USA).

### Lactate Dehydrogenase (LDH) Assay

According to manufacturer protocol, the LDH level in PC-12 cells was measured using the Lactate Dehydrogenase Assay Kit (Abcam). The concentration of LDH (OD450) was analyzed using a Multiskan Spectrum microplate reader (Thermo Fisher Scientific).

### Flow Cytometry Analysis

The apoptosis rate of PC-12 cells was evaluated using the Annexin V-PI apoptosis detection kit (Thermo Fisher Scientific) according to the manufacturer's protocol. Briefly, 5 × 10^5^ cells were resuspended in 500 μl binding buffer. After that, the cells were stained with 5 μl Annexin V-EGFP and PI at 4°C for 15 min in the dark. Subsequently, 400 μl binding buffer was added to the cells and the apoptosis rate of PC-12 cells was assessed using a FACScan flow cytometer (Becton, Dickinson and Company, Franklin lakes, NJ, USA).

### Western Blot Analysis

Following extracting proteins from OGD/R-induced PC-12 cells by RIPA buffer containing protease inhibitors, the BCA Protein Assay Kit (Abcam) was used to detect the protein concentrations. Then, proteins were separated by 10% SDS-PAGE and transferred into PVDF membranes. At room temperature, the blocking was performed using 5% bovine serum albumin (BSA). After blocking, membranes were incubated overnight at 4°C with primary antibodies against Bcl-2 (1:1,000; Abcam), Caspase-3 (1:1,000; Abcam), PDE4D (1:1,000; Abcam), and β-actin (1:1,000; Abcam). Then, tris-buffered saline Tween-20 (TBST) was used to wash the membranes for three times. Subsequently, at room temperature, the secondary antibody HRP-conjugated anti-mouse IgG (1:5,000; Santa Cruz, Waltham, MA, USA) was added to incubate for 1 h. β-Actin was used as the internal reference. The membranes were developed by chemiluminescence reagents (Thermo Fisher Scientific) under a Gel-Pro analyzer (version 4.0, USA).

### Enzyme-Linked Immunosorbent Assay (ELISA)

The levels of the inflammatory factors (TNF-α, IL-1β, and IL-6) in PC-12 cells or brain tissues of rats were measured using specific ELISA kits (Multisciences Biotech, Ltd., Hangzhou, China) according to manufacturer protocol. The Multiskan Spectrum microplate reader (Thermo Fisher Scientific) was used to determine the absorbance at 450 nm.

### Dual Luciferase Reporter (DLR) Assay

The cloning 3′UTR sequences containing the binding site were cloned into the pGL3 vector to establish the wild-type vector. The Phusion Site-Directed Mutagenesis Kit (Thermo Fisher Scientific) was used to construct the mutant-type vector. At 37°C, the wild-type/mutant-type vector and miR-375 mimics/miR-NC were co-transfected into PC-12 cells for 48 h. The Dual-Luciferase Reporter Assay System (Promega) was utilized to detect the relative luciferase activity.

### Statistical Analysis

SPSS software (version 20.0, USA) was used to perform statistical analyses. Data were expressed as the mean ± standard deviation. Student *t*-test was used to assess the differences between two groups. One-way ANOVA was used to evaluate the differences among multiple groups. After ANOVA analysis, the pairwise comparison was performed using Tukey's multiple comparisons test. *P*-value < 0.05 indicated a statistically significant difference. All experiments were conducted in triplicate in at least three independent experiments.

## Results

### MALAT1 Expression Is Upregulated in MCAO/R Models of Rats

**Figure 1:**
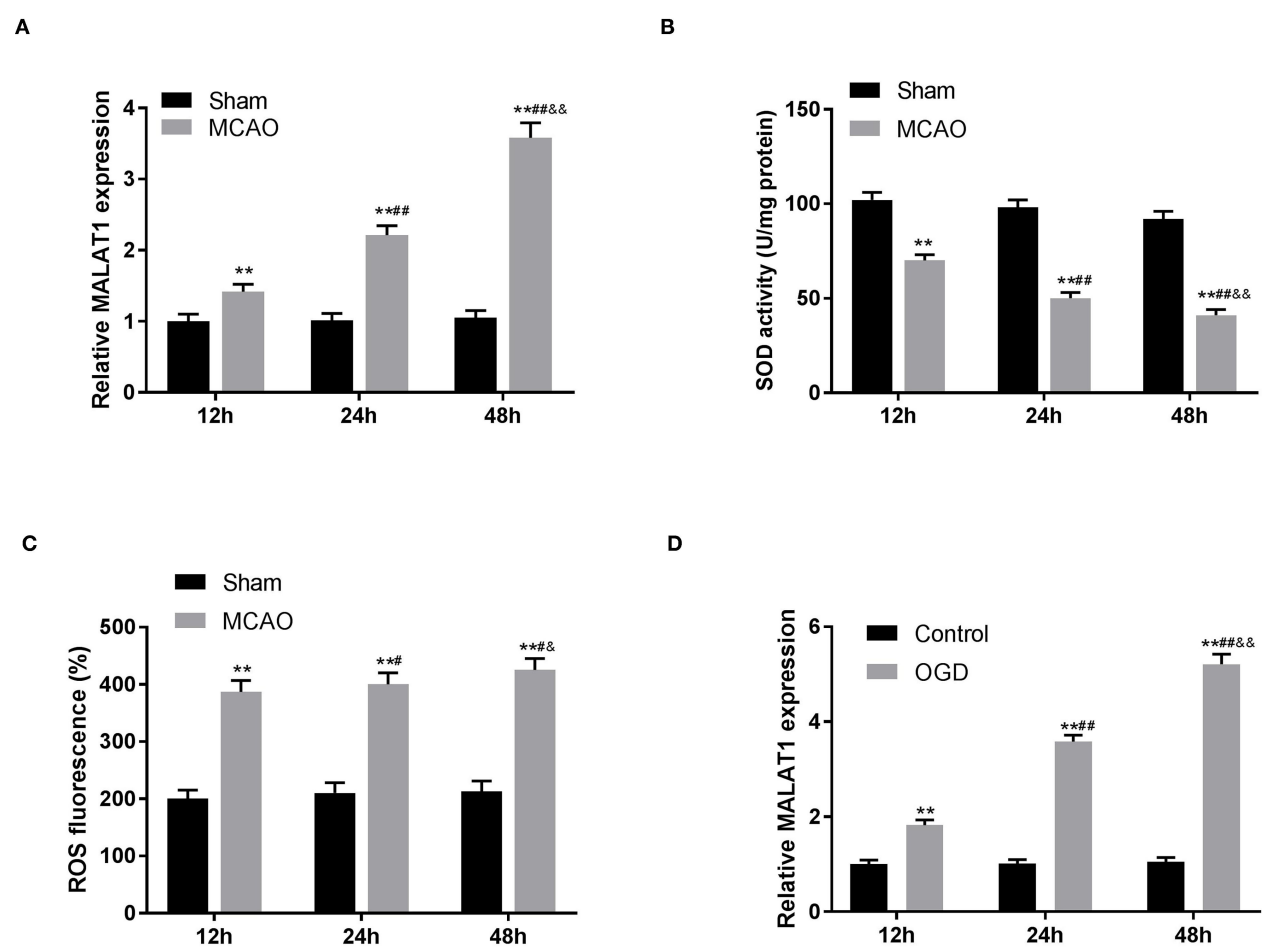
Metastasis-associated lung adenocarcinoma transcript 1 (MALAT1) is highly expressed in middle cerebral artery occlusion (MCAO)/reperfusion (R) models of rats. **(A)** The expression of MALAT1 in brain tissues of rats was detected by quantitative reverse-transcription PCR (qRT-PCR). **P < 0.01 vs. the sham group; ^*##*^*P* < 0.05 vs. the 12 h group; ^&&^*P* < 0.05 vs. the 24 h group. **(B)** The superoxide dismutase (SOD) activity in brain tissues of rats was measured by SOD assay. **P < 0.01 vs. the sham group; ^##^*P* < 0.05 vs. the 12 h group; ^&&^*P* < 0.05 vs. the 24 h group. **(C)** The reactive oxygen species (ROS) level in brain tissues of rats was measured by ROS assay. **P < 0.01 vs. the sham group; ^#^*P* < 0.05 vs. the 12 h group; ^&^*P* < 0.05 vs. the 24 h group. **(D)** The expression of MALAT1 in the oxygen and glucose deprivation/reperfusion (OGD/R) model was detected by qRT-PCR. **P < 0.01 vs. the control group; ^*##*^*P* < 0.05 vs. the 12 h group; ^&&^*P* < 0.05 vs. the 24 h group.

The expression of MALAT1 in brain tissues of rats was detected by qRT-PCR. The results revealed that in contrast to the sham group, MALAT1 expression was distinctly elevated after MCAO/R in a time-dependent manner (Figure 1A, *P* < 0.01). Similarly, in the *in vitro* model (OGD/R), we also found that the expression of MALAT1 was increased in a time-dependent manner (Figure 1D, *P* < 0.01). As presented in Figures 1B,C, the levels of SOD and ROS in brain tissues of rats were measured. The results uncovered that in comparison to the sham group, the SOD level was declined and the ROS level was increased after MCAO/R in a time-dependent manner (Figures 1B,C, *P* < 0.01). Additionally, MALAT1 expression, the levels of SOD and ROS in MCAO/R, and MALAT1 expression in the OGD/R model all had significant differences among the different time points (12, 24, and 48 h) (*P* < 0.05). All the results implied that MALAT1 might be a promoter in CI/RI.

### MALAT1 Inhibition Attenuates OGD/R-Induced Injury of PC-12 Cells

To further investigate the role of MALAT1 on the biological functions of CI/RI *in vitro*, the transfection efficiency of sh-MALAT1-1/-2 or pc-MALAT1 was detected.

**Figure 2:**
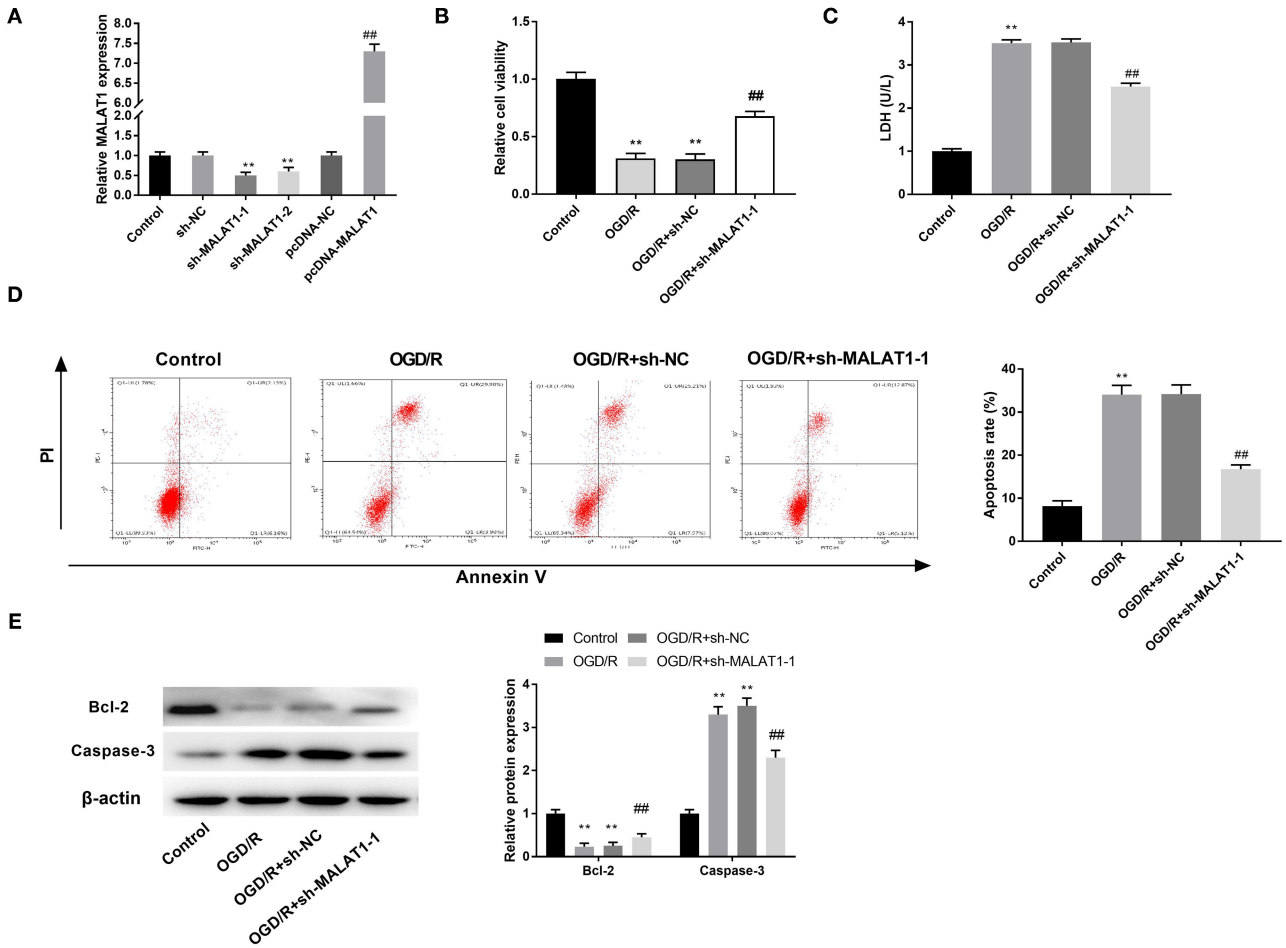
Metastasis-associated lung adenocarcinoma transcript 1 (MALAT1) inhibition attenuates oxygen and glucose deprivation (OGD)/reperfusion (R)-induced injury of PC-12 cells. **(A)** The expression of MALAT1 after transfection of sh-MALAT1-1/-2/NC or pcDNA-NC/MALAT1 into PC-12 cells was detected by quantitative reverse-transcription PCR (qRT-PCR). **P < 0.01 vs. the sh-NC group. ^##^*P* < 0.01 vs. the pcDNA-NC group. **(B)** The viability of OGD/R-induced PC-12 cells was measured by MTT assay. **(C)** The level of lactate dehydrogenase (LDH) in OGD/R-induced PC-12 cells was measured by LDH assay. **(D)** The apoptosis rate of OGD/R-induced PC-12 cells was analyzed by flow cytometry. **(E)** The protein expression of Bcl-2 and Caspase-3 in OGD/R-induced PC-12 cells was measured by Western blot. **P < 0.01 vs. the control group. ^##^*P* < 0.01 vs. the OGD/R group.

The results of qRT-PCR disclosed that MALAT1 expression was downregulated in the sh-MALAT1-1 group or sh-MALAT1-2 group in contrast to the sh-NC group, whereas it was upregulated in the pc-MALAT1 group in comparison to the pc-DNA group (Figure 2A, *P* < 0.01). We chose sh-MALAT1-1 to perform the following experiments due to its relatively high silencing efficiency. MTT assay demonstrated that cell viability in the OGD/R group was significantly restrained compared to the control group. Suppression of MALAT1 reversed the inhibitory effect of OGD/R on cell viability (Figure 2B, *P* < 0.01). LDH assay was utilized to measure the LDH level of PC-12 cells. The results displayed that compared with the control group, the level of LDH was obviously upregulated in the OGD/R group. Suppression of MALAT1 reversed the promoting effect of OGD/R on LDH level (Figure 2C, *P* < 0.01). Flow cytometry analysis revealed that apoptosis rate of PC-12 cells was increased in the OGD/R group in contrast to the control group. Suppression of MALAT1 reversed the promoting effect of OGD/R on apoptosis rate (Figure 2D, *P* < 0.01). Western blot assay was utilized to further measure the protein expression of apoptosis-related factors (Bcl-2 and Caspase-3) in PC-12 cells. We found that in contrast to the control group, OGD/R significantly decreased Bcl-2 protein expression, whereas it increased Caspase-3 protein expression. However, MALAT1 knockdown reversed the inhibitory effect of OGD/R on Bcl-2 protein expression and the promoting effect on Caspase-3 protein expression (Figure 2E, *P* < 0.01). The results suggested that MALAT1 knockdown could attenuate OGD/R injury of PC-12 cells *in vitro*.

### MALAT1 Inhibition Diminishes OGD/R-Induced Inflammatory Response in PC-12 Cells

**Figure 3:**
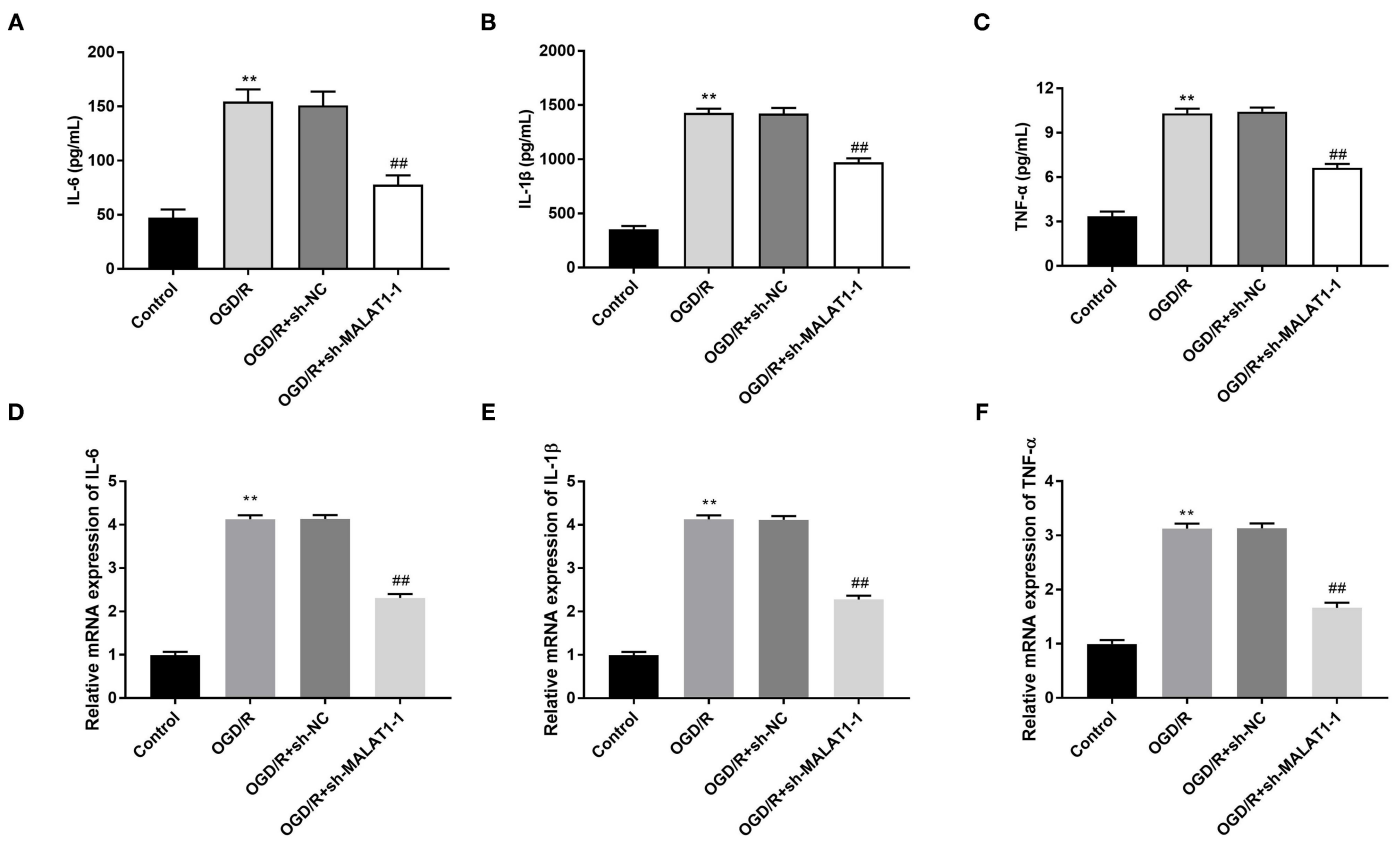
Metastasis-associated lung adenocarcinoma transcript 1 (MALAT1) inhibition diminishes oxygen and glucose deprivation (OGD)/reperfusion (R)-induced inflammatory response in PC-12 cells. **(A)** The level of IL-6 in OGD/R-induced PC-12 cells was measured by enzyme-linked immunosorbent assay (ELISA). **(B)** The level of IL-1β in OGD/R-induced PC-12 cells was measured by ELISA. **(C)** The level of TNF-α in OGD/R-induced PC-12 cells was measured by ELISA. **(D)** The mRNA expression of IL-6 in OGD/R-induced PC-12 cells was detected by quantitative reverse-transcription PCR (qRT-PCR). **(E)** The mRNA expression of IL-1β in OGD/R-induced PC-12 cells was detected by qRT-PCR. **(F)** The mRNA expression of TNF-α in OGD/R-induced PC-12 cells was detected by qRT-PCR. **P < 0.01 vs. the control group. ^*##*^*P* < 0.01 vs. the OGD/R group.

The levels of inflammatory factors (TNF-α, IL-6, and IL-1β) and their mRNAs expression were detected by ELISA and qRT-PCR, respectively. Interestingly, inflammatory factor levels and the mRNA expression were all elevated in the OGD/R group in contrast to the control group. MALAT1 inhibition reversed the promoting effects of OGD/R on the levels of TNF-α, IL-6, and IL-1β and their mRNAs expression (Figures 3A–F, *P* < 0.01). These data implied that secretion of inflammatory factors in OGD/R-induced PC-12 cells could be restrained by MALAT1 knockdown.

### MALAT1 Targets miR-375

**Figure 4:**
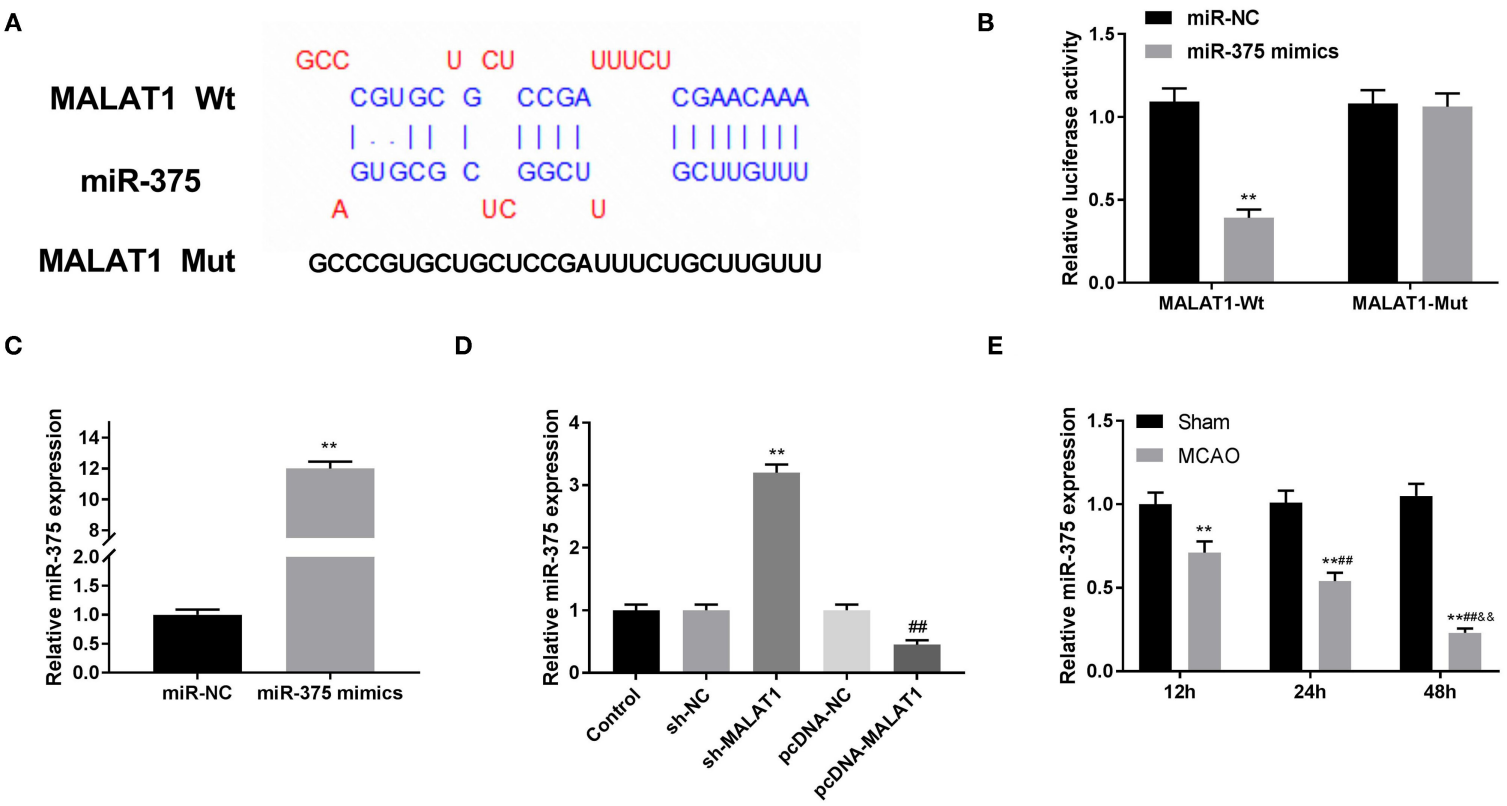
MicroRNA (MiR)-375 is the direct target of metastasis-associated lung adenocarcinoma transcript 1 (MALAT1). **(A)** The predicted complementary binding site of MALAT1 and miR-375. **(B)** The luciferase activity in PC-12 cells co-transfected with pGL3-MALAT1 WT/pGL3-MALAT1 MUT and miR-375 mimics/NC was determined by dual luciferase reporter (DLR) assay. **P < 0.01 vs. the miR-NC group. **(C)** The expression of miR-375 after transfection of miR-375 mimics/NC into PC-12 cells was detected by quantitative reverse-transcription PCR (qRT-PCR). **P < 0.01 vs. the miR-NC group. **(D)** The expression of miR-375 after transfection of sh-MALAT1/NC or pcDNA-NC/MALAT1 into PC-12 cells was detected by qRT-PCR. **P < 0.01 vs. the sh-NC group. ^*##*^*P* < 0.01 vs. the pcDNA-NC group. **(E)** The expression of miR-375 in brain tissues of rats was detected by qRT-PCR. **P < 0.01 vs. the sham group; ^*##*^*P* < 0.05 vs. the 12 h group; ^&&^*P* < 0.05 vs. the 24 h group.

We used the Starbase software to predict the potential binding site between MALAT1 and miR-375 (Figure 4A) and verified the target relationship by DLR assay. The results illustrated that the luciferase activity in the MALAT1-WT/miR-375 mimic group was obviously decreased in contrast to that in the MALAT1-WT/miR-NC group (Figure 4B, *P* < 0.01). Subsequently, qRT-PCR was performed to detect the transfection efficiency of miR-375 mimics in OGD/R-induced PC-12 cells. We found that miR-375 expression in the miR-375 mimic group was highly increased in comparison to the NC mimic group (Figure 4C, *P* < 0.01). To further explore the interactions between MALAT1 and miR-375, sh-MALAT1/pc-MALAT1 was transfected into OGD/R-induced PC-12 cells, respectively. qRT-PCR results revealed that miR-375 expression was upregulated by MALAT knockdown, whereas it was downregulated by MALAT1 overexpression (Figure 4D, *P* < 0.01). Additionally, as shown in Figure 4E, a decreased expression of miR-375 was observed in brain tissues of MCAO/R rats in a time-dependent manner (Figure 4E, *P* < 0.01) and miR-375 expression showed significant differences among the different time points (*P* < 0.01). We speculated that miR-375 was a target of MALAT1 and negatively modulated by MALAT1.

### Overexpression of miR-375 Alleviates OGD/R-Induced Injury of PC-12 Cells

**Figure 5:**
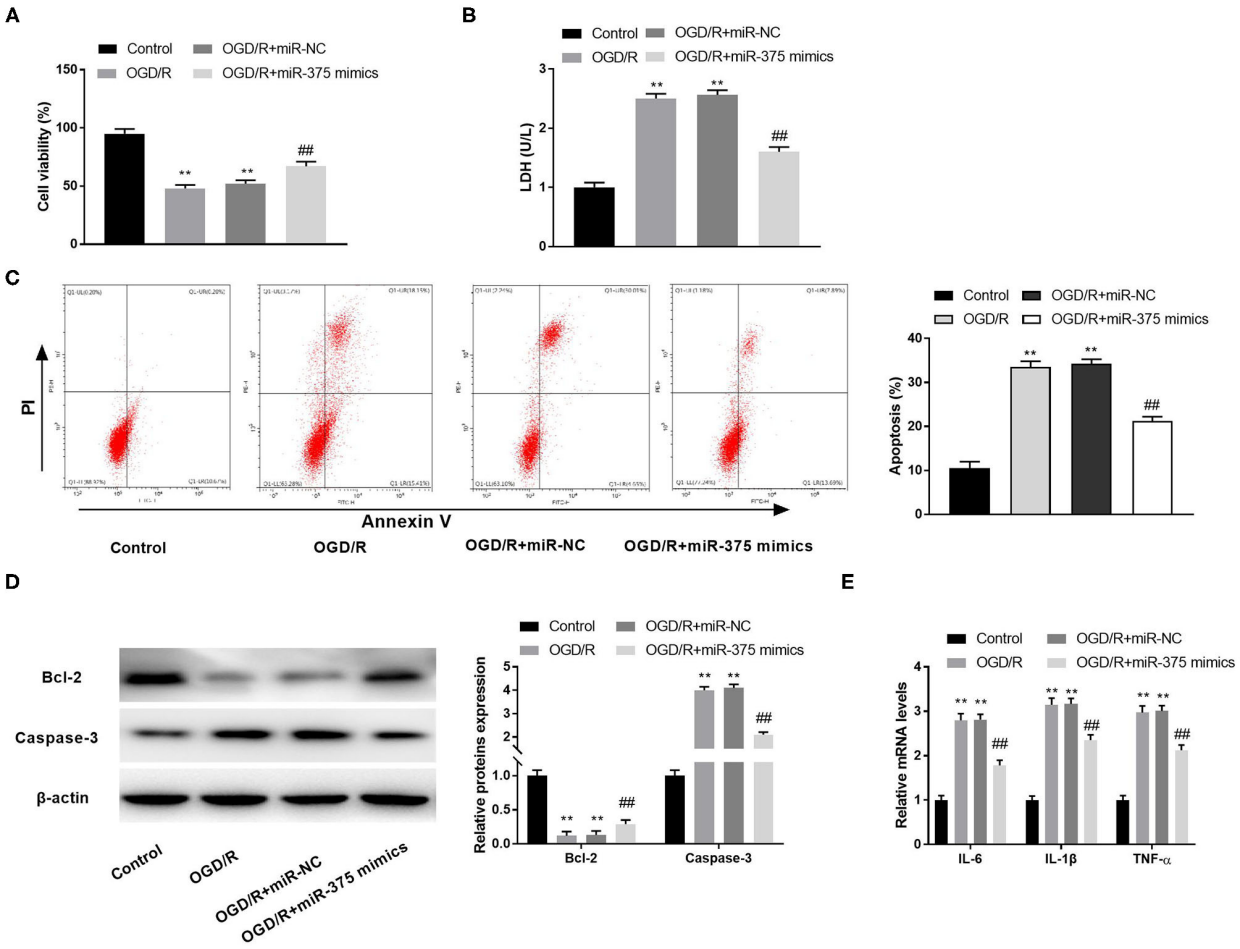
Overexpression of microRNA (miR)-375 alleviates oxygen and glucose deprivation (OGD)/reperfusion (R)-induced injury of PC-12 cells. **(A)** The viability of OGD/R-induced PC-12 cells was measured by MTT assay. **(B)** The level of lactate dehydrogenase (LDH) in OGD/R-induced PC-12 cells was measured by LDH assay. **(C)** The apoptosis rate of OGD/R-induced PC-12 cells was analyzed by flow cytometry. **(D)** The protein expression of Bcl-2 and Caspase-3 in OGD/R-induced PC-12 cells was measured by Western blot. **(E)** The mRNA levels of IL-6, IL-1β, and TNF-α in OGD/R-induced PC-12 cells were detected by quantitative reverse-transcription PCR (qRT-PCR). **P < 0.01 vs. the control group. ^##^*P* < 0.01 vs. the OGD/R group.

To investigate the possible role of miR-375 mimics on CI/RI *in vitro*, we first measured cell viability in the OGD/R model. The results of MTT assay disclosed that OGD/R dramatically decreased the cell viability, while miR-375 overexpression rescued the cell viability (Figure 5A, *P* < 0.01). LDH assay and flow cytometry analysis, respectively, uncovered that the LDH level or apoptosis rate of PC-12 cells was elevated by OGD/R. Interestingly, overexpression of miR-375 reversed the promoting effect of OGD/R on the LDH level and apoptosis rate of PC-12 cells (Figures 5B,C, *P* < 0.01). Subsequently, the protein expression of apoptosis-related factors (Bcl-2 and Caspase-3) in OGD/R-induced PC-12 cells was measured by western blot assay. We discovered that OGD/R significantly decreased Bcl-2 protein expression and increased Caspase-3 protein expression in contrast to the control. Nevertheless, overexpression of miR-375 reversed the inhibitory effect of OGD/R on Bcl-2 protein expression and the promoting effect on Caspase-3 protein expression (Figure 5D, *P* < 0.01). We also demonstrated that the mRNA levels of inflammatory factors (TNF-α, IL-6, and IL-1β) were all elevated in the OGD/R group. Overexpression of miR-375 reversed the promoting effect of OGD/R on the mRNA levels of inflammatory factors (Figure 5E, *P* < 0.01). The results suggested that miR-375 might be a protector in OGD/R-induced PC-12 cells.

### MiR-375 Targets PDE4D

**Figure 6:**
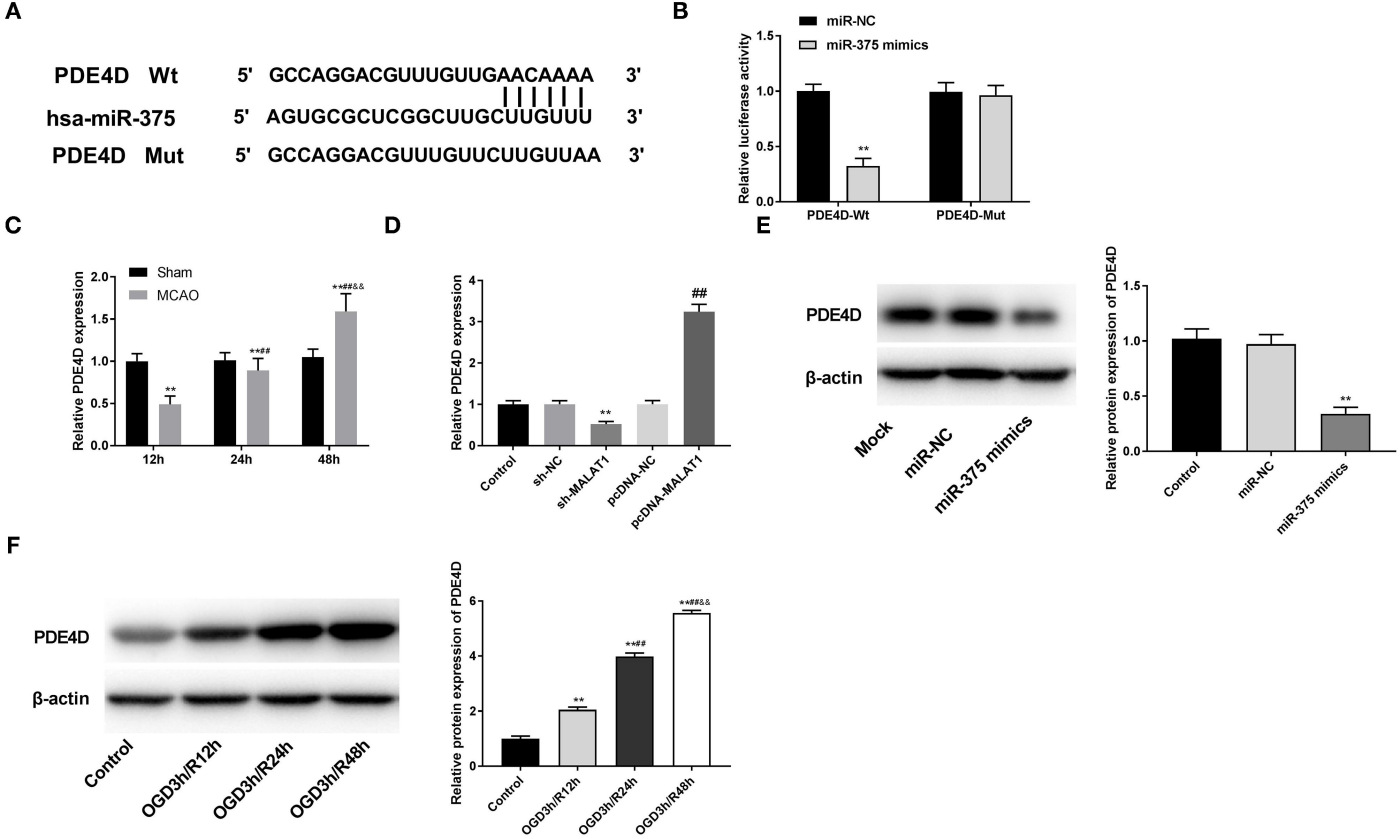
MicroRNA (MiR)-375 targets phosphodiesterase 4D (PDE4D). **(A)** The predicted complementary binding site of miR-375 and PDE4D. **(B)** The luciferase activity in PC-12 cells co-transfected with pGL3-PDE4D WT/pGL3-PDE4D MUT and miR-375 mimics/NC was determined by dual luciferase reporter (DLR) assay. **P < 0.01 vs. the miR-NC group. **(C)** The expression of PDE4D in brain tissues of rats was detected by quantitative reverse-transcription PCR (qRT-PCR). **P < 0.01 vs. the sham group; ^##^*P* < 0.05 vs. the 12 h group; ^&&^*P* < 0.05 vs. the 24 h group. **(D)** The expression of PDE4D after transfection of sh-MALAT1/NC or pcDNA-NC/MALAT1 into PC-12 cells was detected by qRT-PCR. **P < 0.01 vs. the sh-NC group. ^##^*P* < 0.01 vs. the pcDNA-NC group. **(E)** The protein expression of PDE4D after transfection of miR-375 mimics/NC into PC-12 cells was measured by Western blot. **P < 0.01 vs. the miR-NC group. **(F)** The protein expression of PDE4D in OGD/R-induced PC-12 cells was measured by Western blot. **P < 0.01 vs. the control group; **P < 0.01 vs. the control group; ^##^*P* < 0.05 vs. the OGD3h/R12 h group; ^&&^*P* < 0.05 vs. the OGD3h/R24 h group.

Through Targetscan software, the potential binding site between miR-375 and PDE4D was predicted (Figure 6A). DLR assay revealed that in comparison to the PDE4D-WT/miR-NC group, the luciferase activity in the PDE4D-WT/miR-375 mimic group was declined (Figure 6B, *P* < 0.01). Meanwhile, compared to the Sham group, we found that PDE4D expression was increased in brain tissues of rats in the MCAO group in a time-dependent manner (Figure 6C, *P* < 0.01). To explore whether MALAT1 alters the expression of PDE4D, sh-MALAT1/pc-MALAT1 was transfected into OGD/R-induced PC-12 cells to detect PDE4D expression. The results of qRT-PCR demonstrated that PDE4D expression was downregulated by sh-MALAT1, whereas it was upregulated by pcDNA-MALAT1 (Figure 6D, *P* < 0.01). In addition, to further determine the interaction between miR-375 and PDE4D, PDE4D protein expression was detected by western blot assay after transfection of miR-375 mimics/NC mimics into OGD/R-induced PC-12 cells. The results illustrated that PDE4D protein expression was diminished by miR-375 overexpression (Figure 6E, *P* < 0.01). Interestingly, the protein expression of PDE4D was elevated by OGD/R in a time-dependent manner (Figure 6F, *P* < 0.01). Furthermore, as illustrated in Figures 6C,F, both PDE4D expression in brain tissues of rats or PDE4D protein level in the OGD/R model presented significant differences among the different time points (*P* < 0.05). The data implied that PDE4D might be a promoter in OGD/R-induced PC-12 cells. PDE4D was the target gene of miR-375 and negatively modulated by miR-375.

### Upregulation of miR-375 Ameliorates OGD/R-Induced Injury of PC-12 Cells Through Modulating PDE4D

**Figure 7:**
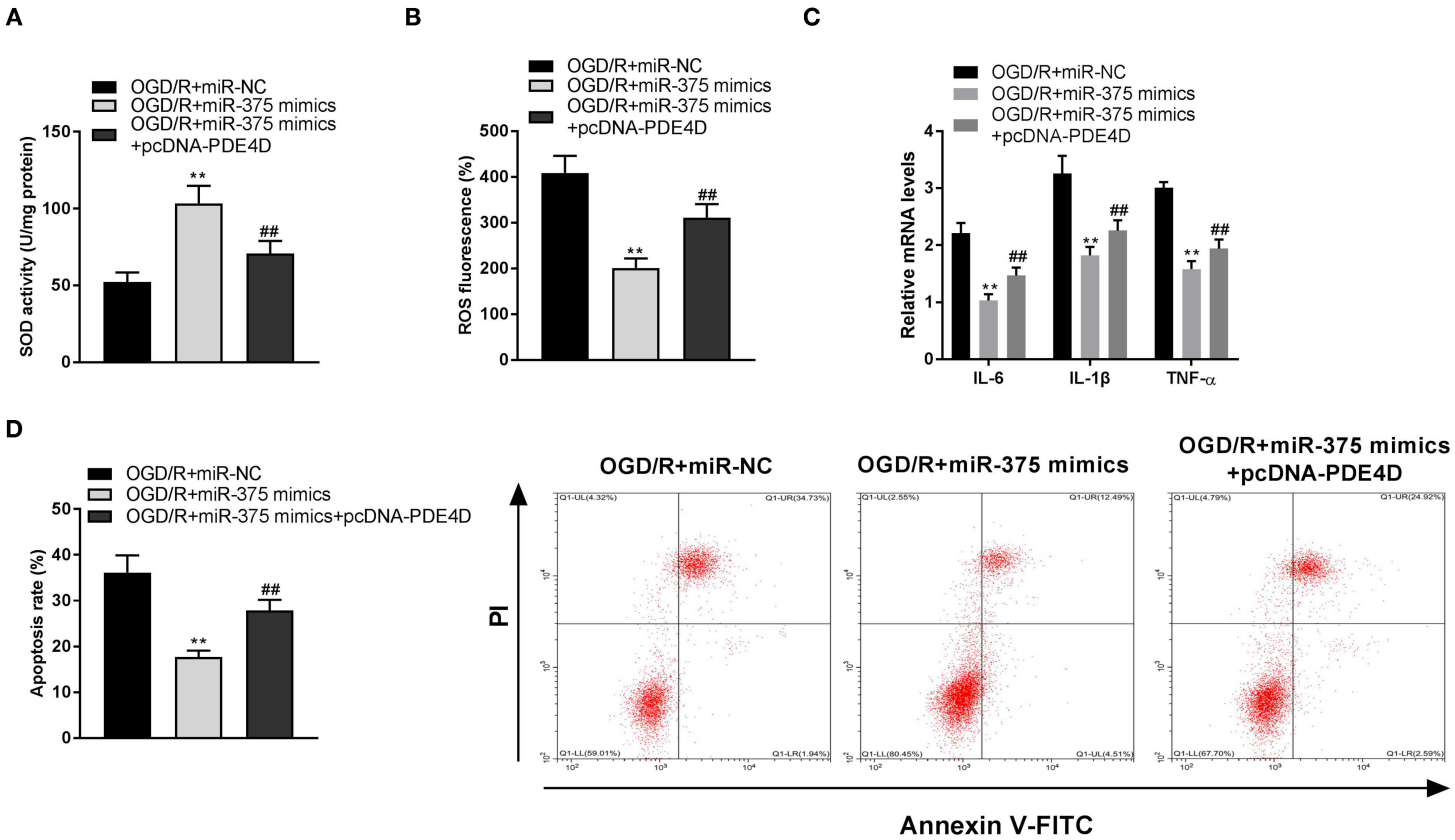
Upregulation of microRNA (miR)-375 ameliorates oxygen and glucose deprivation (OGD)/reperfusion (R)-induced injury of PC-12 cells through modulating phosphodiesterase 4D (PDE4D). **(A)** The superoxide dismutase (SOD) activity in OGD-induced PC-12 cells was measured by SOD assay. **(B)** The reactive oxygen species (ROS) level in OGD-induced PC-12 cells was measured by ROS assay. **(C)** The mRNA levels of IL-6, IL-1β, and TNF-α in OGD/R-induced PC-12 cells were detected by quantitative reverse-transcription PCR (qRT-PCR). **(D)** The apoptosis rate of OGD/R-induced PC-12 cells was analyzed by flow cytometry. **P < 0.01 vs. the OGD/R + miR-NC group. ^##^*P* < 0.01 vs. the OGD/R + miR-375 mimics group.

To investigate the regulatory mechanism of the miR-375/PDE4D axis on OGD/R-stimulated injury *in vitro*, rescue experiments were performed. SOD detection assay showed the results that the SOD level of OGD/R-induced PC-12 cells transfected with miR-375 mimics was enhanced (Figure 7A, *P* < 0.01), and this situation was partly suppressed by transfection of pcDNA-PDE4D (Figure 7A, *P* < 0.01). As illustrated in Figures 7B–D, we further discovered that the inhibitory effects of miR-375 upregulation on ROS levels, mRNA levels of inflammatory factors (TNF-α, IL-6, and IL-1β), and apoptosis rate were reversed by overexpression of PDE4D (*P* < 0.01).

### MALAT1 Knockdown Alleviates OGD/R Injury of PC-12 Cells Through Regulating miR-375/PDE4D

**Figure 8:**
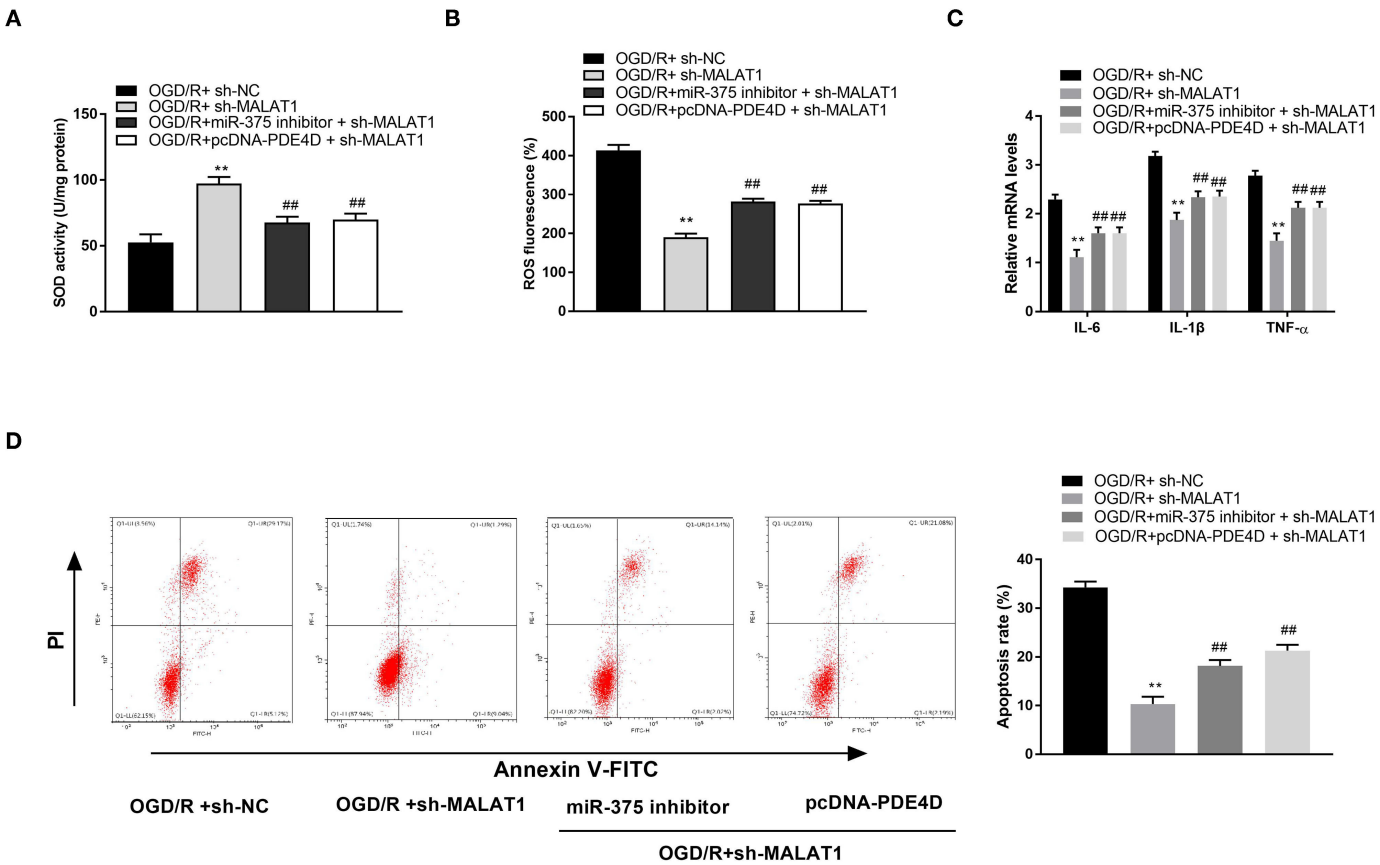
Metastasis-associated lung adenocarcinoma transcript 1 (MALAT1) knockdown alleviates oxygen and glucose deprivation (OGD)/reperfusion (R)–induced injury of PC-12 cells through regulating microRNA (miR)-375/phosphodiesterase 4D (PDE4D). **(A)** The superoxide dismutase (SOD) activity in OGD-induced PC-12 cells was measured by SOD assay. **(B)** The reactive oxygen species (ROS) level in OGD-induced PC-12 cells was measured by ROS assay. **(C)** The mRNA levels of IL-6, IL-1β, and TNF-α in OGD/R-induced PC-12 cells were detected by quantitative reverse-transcription PCR (qRT-PCR). **(D)** The apoptosis rate of OGD/R-induced PC-12 cells was analyzed by flow cytometry. **P < 0.01 vs. the OGD/R + sh-NC group. ^*##*^*P* < 0.01 vs. the OGD/R + sh-MALAT1 group.

After further transfection of miR-375 inhibitor + sh-MALAT1 or pcDNA-PDE4D + sh-MALAT1 into OGD/R-induced PC-12 cells, the feedback verification experiments were performed. We found that the SOD level was elevated by MALAT1 knockdown, whereas both downregulation of miR-375 and upregulation of PDE4D reversed the promoting effect of MALAT1 on the level of SOD (Figure 8A, *P* < 0.01). As shown in Figures 8B–D, ROS level, mRNA levels of inflammatory factors (TNF-α, IL-6, and IL-1β), and apoptosis rate of PC-12 cells were all restrained by MALAT1 inhibition. However, both downregulation of miR-375 and upregulation of PDE4D reversed the suppressive effects of MALAT1 on the ROS level, mRNA levels of inflammatory factors (TNF-α, IL-6, and IL-1β), and apoptosis rate. We conjectured that MALAT1 knockdown alleviated OGD/R injury of PC-12 cells through regulating miR-375/PDE4D *in vitro*.

## Discussion

CI/R contributes to irreversible damage to brain tissues and cells, which severely affects the health and life of cerebral IS patients (6). It is generally recognized that use of the MCAO/R model from mice or rats has great significance in clinic, which promotes the development of medical research on human CI/RI (6, 24, 25). In this study, the MCAO/R model was utilized to detect MALAT1 expression in brain tissues of CI/RI rats. Numerous studies have reported that lncRNAs may serve as a regulator in CI/RI (6, 8). For instance, AK038891 is highly expressed in a MCAO/R model of rat (6). Zhong et al. have uncovered that the expression of SNHG14 is distinctly upregulated in brain tissues of a MCAO/R rat model (8). Consistent with the above results, we discovered that in the MCAO/R rat model, MALAT1 expression in brain tissues of rats was elevated in a time-dependent manner. In addition, we further verified that the levels of SOD and ROS in brain tissues of rats were decreased and increased, respectively. Therefore, our results implied that MALAT1 might be a promotor to result in injury of CI/R.

Increasing data have been reported that in OGD/R-induced cells, aberrant expression of lncRNAs is participated in modulating I/R-related cellular behaviors, such as cell viability (8), inflammatory factors release (25), generation of LDH (8), and programmed cell death (25). Zhong et al. have disclosed that SNHG14 inhibition reverses the inhibiting effect of OGD/R on cell viability and the promoting effect on LDH level (8). Shangguan et al. have illustrated that OGD/R treatment promotes the apoptosis rate of cells and secretion of inflammatory factors, whereas knockdown of GAS5 reverses these effects (25). Consistent with the above two researches, we found that downregulation of MALAT1 reversed the inhibitory effect of OGD/R on cell viability and the promoting effects on LDH level, inflammatory-factor levels, and apoptosis rate of cells. The results implied that MALAT1 knockdown attenuated the injury of OGD/R in PC-12 cells. Similarly, Tian et al. have reported that MALAT1 knockdown decreases inflammatory-factor release in CoCl_2_-induced HK2 cells. However, the previous study only investigated the effect of MALAT1 knockdown on the levels of inflammatory factors. Our results further confirmed that knockdown of MALAT1 was involved in regulating cell viability, apoptosis, and LDH level. Therefore, we conjectured that MALAT1 knockdown functioned as a neuroprotective role against OGD/R injury *in vitro*.

Previous researches have been indicated that miRNAs exhibit their neuroprotective roles against I/R injury, especially in OGD/R-induced cells (14, 26, 27). Lu et al. have confirmed that miR-219a-5p can suppress OGD/R-induced cell apoptosis and LDH release in mouse neuroblastoma cells (14). Chen et al. have uncovered that after OGD/R treatment, miR-1306-5p upregulation elevates cell viability and restrains LDH level and cell apoptosis (27). A recent study conducted by Ou et al. has verified that miR-375 targeting Ctgf provides significant protection from injury of cerebral I/R, which is reflected by reduced infarct volumes and cell apoptosis, increased proliferation, and migration of PC12 cells (15). Similarly, in this study, we illustrated that in OGD/R-induced PC-12 cells, overexpression of miR-375 promoted cell viability, whereas it inhibited cell apoptosis. Additionally, we further demonstrated that upregulation of miR-375 had inhibiting effects on the levels of LDH and inflammation. The results implied that miR-375 could attenuate injury of OGD/R in PC-12 cells. Besides, miR-375 has been confirmed to be a target of MALAT1 and was negatively modulated by MALAT1. We inferred that MALAT1 was involved in OGD/R injury through modulating miR-375. To further verify this assumption, we performed the feedback verification experiment between MALAT1 and miR-375 in OGD/R-induced PC-12 cells. We demonstrated that downregulation of miR-375 reversed the promoting effect of MALAT1 knockdown on SOD level and the suppressive effects on ROS level, inflammatory factor levels, and cell apoptosis. Therefore, the above results implied that knockdown of MALAT1 alleviated OGD/R injury in PC-12 cells through modulating miR-375.

Enzymes within the PDE family are found in the central nervous system (28) and most pro-inflammatory and immune cells (16). As a member of the PDE family, phosphodiesterase 4D (PDE4D) is confirmed to highly express in OGD/R-induced cells (14). Consistently, we uncovered that after OGD treatment, PDE4D protein expression was increased with the extension of reperfusion time. The results hinted that PDE4D might be a promotor involved in the progression of OGD/R. In addition, PDE4D has been reported to take part in several cellular processes, such as inflammatory factor release (28, 29) and cell apoptosis (30). Similarly, our study illustrated that overexpression of PDE4D reversed the promoting effect of MALAT1 knockdown on SOD level and the inhibitory effects on ROS level, inflammatory factor levels, and cell apoptosis. The results suggested that MALAT1 knockdown alleviated OGD/R injury in PC-12 cells through modulating PDE4D. Besides, PDE4D was the target gene of negative regulation by miR-375. The rescue experiments between miR-375 and PDE4D uncovered a fact that miR-375 ameliorated OGD/R injury via mediating PDE4D expression *in vitro*. We further drew a conclusion that MALAT1 knockdown alleviated OGD/R injury in PC-12 cells through modulating the miR-375/PDE4D axis.

In summary, the current study uncovered that MALAT1 was upregulated in a MCAO/R model of rat, whereas MALAT1 knockdown ameliorated OGD/R injury by regulating the miR-375/PDE4D axis *in vitro*. We speculated that MALAT1 might be a new target for treating CI/RI. However, this study did not confirm the detailed mechanisms among MALAT1, miR-375, and PDE4D *in vivo*, which may be a limitation of the present study. Further investigations are still required to confirm our findings.

## Data Availability Statement

The original contributions presented in the study are included in the article/supplementary material, further inquiries can be directed to the corresponding author/s.

## Ethics Statement

The animal study was reviewed and approved by Laboratory Animal Ethics Committee of Weifang People's Hospital.

## Author Contributions

GZ, QW, and DS: conception and design, analysis of data, and drafting of the article. GZ, QW, DS, and YX: carrying out of the experiment. GZ and YX: critical revision of the article for important intellectual content. All authors: approved the manuscript.

## Conflict of Interest

The authors declare that the research was conducted in the absence of any commercial or financial relationships that could be construed as a potential conflict of interest.
